# Exploring the “Latin American Mediterranean” family and the RD^Rio^ lineage in *Mycobacterium tuberculosis* isolates from Paraguay, Argentina and Venezuela

**DOI:** 10.1186/s12866-019-1479-6

**Published:** 2019-06-13

**Authors:** Chyntia Carolina Díaz Acosta, Graciela Russomando, Norma Candia, Viviana Ritacco, Sidra E. G. Vasconcellos, Marcia de Berrêdo Pinho Moreira, Nilda J. de Romero, Nora Morcillo, Jacobus Henri De Waard, Harrison Magdinier Gomes, Philip Noel Suffys

**Affiliations:** 1 Departamento de Biología Molecular y Biotecnología. Instituto de Investigaciones en Ciencias de la Salud, Universidad Nacional de Asunción, Asunción, Paraguay; 2 Laboratório de Biologia Molecular aplicada às Micobactérias, Instituto Oswaldo Cruz, Fundação Oswaldo Cruz, Rio de Janeiro, RJ 21045-900 Brazil; 3 Servicio de Micobacterias, Instituto Nacional de Enfermedades Infecciosas, ANLIS “Carlos G. Malbran”, Buenos Aires, Argentina; 4 Laboratório de Microbiologia Celular, Instituto Oswaldo Cruz, Fundação Oswaldo Cruz, Rio de Janeiro, Brazil; 5 Laboratorio Central de Salud Pública, MSP y BS, Asunción, Paraguay; 6 Instituto Nacional de Enfermedades Respiratorias Emilio Coni, Buenos Aires, Argentina; 7 Laboratorio de Tuberculosis, Instituto de Biomedicina, Caracas, Venezuela; 8 Present Address: One Health Research Group. Facultad de Ciencias de la Salud, Universidad de Las Américas (UDLA), Quito, Ecuador

**Keywords:** *Mycobacterium tuberculosis*, LAM, Genotyping, Paraguay, South-America

## Abstract

**Background:**

The Latin American & Mediterranean (LAM) spoligotype family is one of the most successful genotype of *Mycobacterium tuberculosis* worldwide and particularly prevalent in South-America. Within this family, a sublineage named Region of Difference Rio (RD^Rio^) was reported initially in Brazil and is characterized by a genomic deletion of about 26.3 kb. This lineage seems to show a specific adaptation to the Euro-Latin American population. In this context, we sought to evaluate the LAM family and the presence of the RD^Rio^ genotype in samples from three Latin American countries including Paraguay, Venezuela and Argentina. To detect LAM strains reliably we applied a typing scheme using spoligotyping, 12 loci MIRU-VNTR, the Ag85C^103^ SNP and the regions of difference RD^Rio^ and RD174. IS6110-RFLP results were also used when available.

**Results:**

Genotyping of 413 *M. tuberculosis* isolates from three Latin-American countries detected LAM (46%) and the ill-defined T clade (16%) as the most frequent families. The highest clustering rate was detected in the sample population from the city of Caracas in Venezuela. We observed considerable differences in the presence of the RD^Rio^ lineage, with high frequency in Caracas-Venezuela (55%) and low frequency in Buenos Aires-Argentina (11%) and Paraguay (10%). The molecular markers (RD174, Ag85C^103^, MIRU02-MIRU40 signature) of the RD^Rio^ lineage were essentially confirmed. For the LAM family, the most polymorphic loci were MIRU40, MIRU31, MIRU10, MIRU26, MIRU16 and the least polymorphic MIRU24, MIRU20, MIRU04, MIRU23.

**Conclusions:**

Our results suggest a differential adaptation of LAM-sublineages in neighboring populations and that RD^Rio^ strains spread regionally with different rates of distribution. The Ag85C SNP and RDs (RD174, RD^Rio^) tested in this study can in fact facilitate molecular epidemiological studies of LAM strains in endemic settings and low-income countries.

**Electronic supplementary material:**

The online version of this article (10.1186/s12866-019-1479-6) contains supplementary material, which is available to authorized users.

## Background

Tuberculosis (TB) is one of the leading causes of morbidity and mortality in humans. In 2017, there were an estimated 10 million incident cases of TB globally, equivalent to 142 cases per 100,000 individuals [1]. Screening with molecular genotyping methods has identified numerous genotypes and lineages of *M. tuberculosis* (Mtb) which led us to a better understanding of the genetic differences between strains. Studies that monitor the dynamics in the population structure of Mtb are highly relevant and in conjunction with classical epidemiological investigations constitute powerful tools for TB surveillance at national, regional, and global levels [2, 3]. In fact, over time, molecular data has allowed us to observe a phylogeographically structured global epidemic, result of longstanding and ongoing evolution [4]. Furthermore, genetic variation has been shown to impact on pathogenicity, virulence, transmissibility or the ability to subvert host immune responses [5–10]. Publicly available databases such as SITVITWEB [11] and MIRU-VNTRplus [2] allow comparison of TB multimarker-based genotyping data and global epidemiological studies [11, 12]. According to SITVITWEB, the “Latin American-Mediterranean” (LAM) is highly prevalent globally [11] and regionally it constitutes an endemic pattern [13–19]. Although diversity in spoligotype and IS*6110-*RFLP patterns have been used extensively as indicators of overall genomic differentiation in Mtb [20, 21] nowadays, MIRUs, SNPs and LSPs represent robust markers for strain classification and for inferring phylogenies [22, 23]. In fact, the SNP *fbpC*^*103*^ in codon 103 (G to A) of the gene encoding antigen 85 Complex C (Ag85C or Rv0129c) has been described as a robust LAM family marker [24]. Within the LAM family, a large sequence polymorphism named Region of Difference Rio (RD^Rio^) has been first detected in Brazil [25]. Recent data showed RD^Rio^ to be present in many countries on different continents [15, 26–28] and in some settings, it was associated with multidrug resistance [29] or with higher levels of recent transmission. The RD^Rio^ lineage is strictly associated with the Euro-Latin American *Mtb* isolates and predominantly associated to certain LAM strains [25]. LAM1 and LAM2 were reported to be exclusively of the RD^Rio^ genotype, whereas LAM3 was solely represented in the WT genotype, and LAM4, LAM5, LAM6, LAM9 were represented in both the RD^Rio^ and WT LAM genotypes [25]. Co-markers of RD^Rio^ strains were proposed to be the RD174 deletion and two and a single copy of MIRU02 and MIRU40 respectively [24, 25]. Although, RD174 seems to have occurred earlier in evolution [30]. To understand the different capacities of lineages to transmit, to adapt or to co-evolve and eventually cause disease in determined human populations, more studies are necessary. Because of the high frequency and diversity of LAM strains in South America, we performed a cross-sectional study to evaluate the behavior of the LAM and the RD^Rio^ genotypes, in samples from Paraguay as well as in samples from nearby Buenos Aires Province (Argentina) and the distant City of Caracas (Venezuela) for comparison. In 2017, the Paraguayan National TB Program registered 2770 cases of TB in all forms, with a reported incidence rate of 44/100,000. A total of 2579 notifications were new cases and relapses [1]. But the reported incidence rate is not homogeneous in terms of geographical distribution. Very high rates (> 84/100,000) are found in the Chaco Region (Alto Paraguay, Boquerón, Presidente Hayes), high rates (50–84/ 100,000) in Central and Amambay Sanitary Regions; a low incidence rate (< 24/100,000) was found in five Sanitary Regions of the southeast (Ñeembucú, Misiones, Cordillera, Caaguazú and Itapúa) while a moderate rate was notified in the rest of the sanitary regions [31]. Even though, the Chaco Region has a high rate, it is a sparsely populated accounting for 60% of the Paraguayan territory and only about 2% of the total 6.8 million population. In fact, Central, Asunción, Alto Paraná and Presidente Hayes Sanitary regions represent 60% of the country’s disease burden [31]. The present study was undertaken with Mtb strains from different regions of South-America in order to shed some light on differential adaptation of LAM strains. We also sought to assess the usefulness of different genetic markers to detect the LAM family at a regional and country-based level.

## Methods

### Clinical *Mtb* isolates

Clinical isolates for this study were obtained from cohorts from three countries. Samples were mainly from Paraguay. Samples from Venezuela and Argentina were added for comparison purpose. For the Paraguayan cohort, a strain library established at the institute (IICS) was used. Strains were isolated by the national TB reference laboratory network mainly from sputum but also from other body fluids (bronchoalveolar lavage, cerebrospinal fluid, pleural effusion, gastric juice) after culture on Löwenstein-Jensen. The cohort consist of two groups, a first one containing 151 samples that had been previously submitted to spoligotyping and *IS6110*-RFLP as reported by [18] and were obtained during the first national drug resistance surveillance. The second group (*n* = 112) has been collected between 2005 and 2007 for routine diagnosis purpose by the national TB reference laboratory network. The strains form Argentina, were isolated in the Buenos Aires province. A total of 56 strains were obtained from a strain library at the Hospital “Cetrángolo de Vicente López”. Clinical samples collected consecutively for diagnosis purpose in 2005 were cultured using BACTEC™ MGIT™ 960 Mycobacterial Detection System (BD, USA). For the cohort from Venezuela, a total of a 100 isolates were obtained from a strain library at the “Laboratorio de Tuberculosis, Instituto de Biomedicina”. Samples were collected for diagnosis purpose in 2007 at the University Hospital Vargas (San José, Caracas).

### Genotyping

Isolates were submitted to DNA extraction by the cetyl-trimethyl ammonium bromide (CTAB) protocol [32]. Strains were classified as RDRio by multiplex PCR [25] and screened for the genetic signatures of the RD^Rio^ [24]. The 12 loci-MIRU VNTR typing was carried out manually by allele scoring [23]. Control DNA samples with known MIRU profile were included for PCR and electrophoresis quality control. Spoligotyping was performed by reverse hybridization as described previously [21, 33] using commercially available membranes (Ocimum Biosolutions, Hyderabad, India).

### Computer analysis

The spoligotype patterns were compared with the SITVITWEB international database of the Pasteur Institute of Guadalupe (http://www.pasteur-guadeloupe.fr:8081/SITVITDemo online version accessed in December 2017) to determine the Spoligotype International Type (SIT), family and international location [11]. The patterns were also classified using Spotclust [34]. The MIRU-VNTR patterns were analyzed using the SITVITWEB for MIRU International Type (MIT) determination and MIRU-VNTR*plus* for grouping [11, 35]. For similarity search combining both spoligotyping and MIRU-VNTR the categorical similarity coefficient was used applying the same weight for both techniques, and the priority rule was based on Single Locus Variants. The loss or gain of one or multiple copies in MIRU loci was considered as an equally probable event for the construction of the Multiple Spanning tree (MST). Significance of association between genetic markers was assessed using the Pearson’s Chi square Test (*p* < 0.05).

### Ethics approval and consent to participate

The strains analyzed for this study were those routinely obtained as requested by the National Tuberculosis Programs in each country for diagnosis or treatment follow-up. All laboratory specimens were identified by a coded number, and they were handled blindly without the possibility to disclose patient’s identity. For genotyping of mycobacterial strains no informed consent was needed. This study was approved by the Scientific and Ethics Committee of the Institute of Research in Health Sciences (IICS), National University of Asunción by process number P7/03. In Paraguay the current regulation for bioethics is set by the National Bioethics Commission created by Executive Resolution N° 438/2017. In Buenos Aires, when sample collection was carried out, Cetrangolo Hospital had a general informed consent form for both ambulatory or in patients that was used according with the medical procedures. In this form it was specified that the clinical specimens obtained would be used with research purposes. No clinical or personal data of patients from Buenos Aires were used. The Research Committee approved research before starting it. Legislation that rules in Buenos Aires Province for Ethics in research is Law N° 11,044 and its application from the Ministry of Health by Resolution N° 4107/2009. The strains from Caracas, Venezuela were isolated for routine diagnosis purpose in the year 2007 in the “Tuberculosis Laboratory” in Hospital “Dr. Jose Maria Vargas de Caracas”. Bacterial strains were coded and send to the Laboratory of Tuberculosis (Instituto de Biomedicina) for genotyping. It is impossible to identify patients from the strains. Moreover, no clinical or personal data of the patients have been used for the publication. Bioethics regulation in Venezuela is set by the Código de bioética y bioseguridad. Ministerio de Ciencia y Tecnología. Fondo Nacional de Ciencia, Tecnología e Innovación.

## Results

### Spoligotyping

**Fig. 1:**
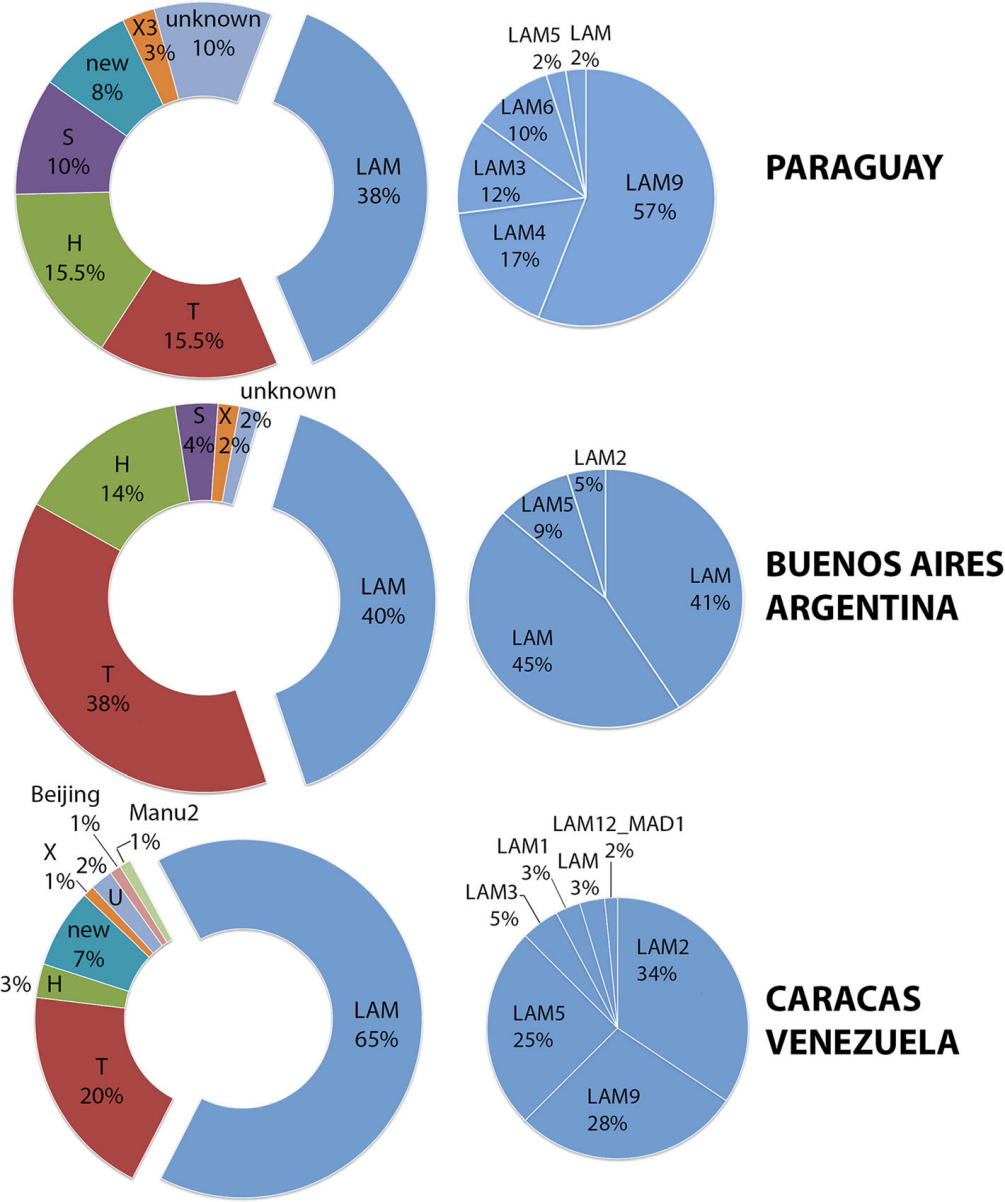
Frequency of distribution of spoligotyping families in each study population. The LAM family subtypes are shown in the blue pie charts

**Fig. 2:**
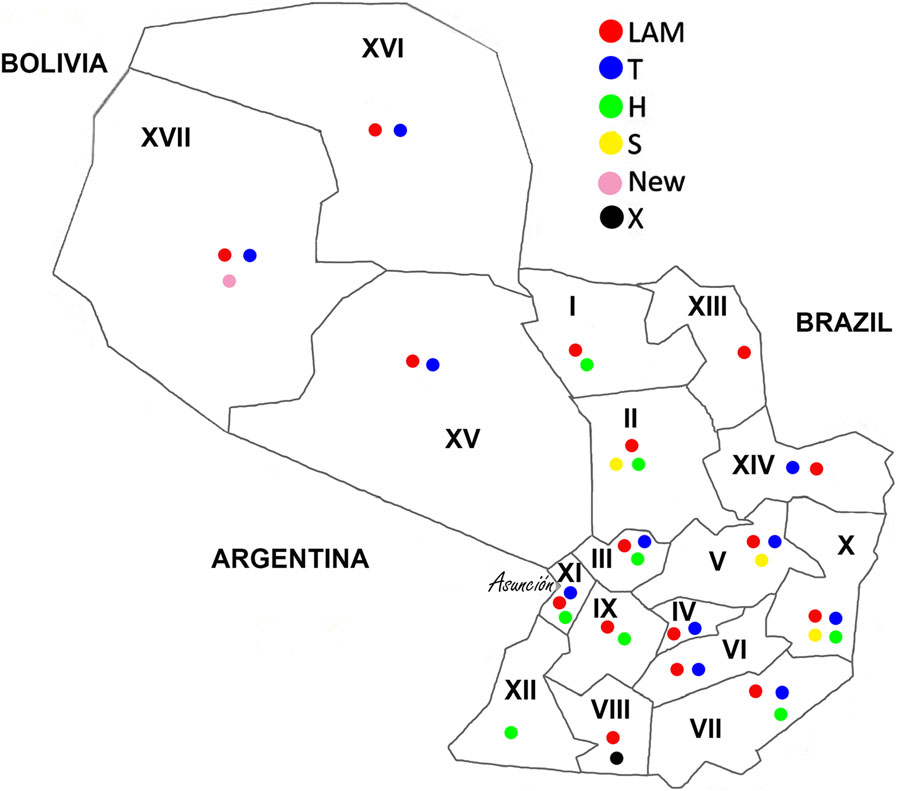
Distribution of the most frequent spoligotyping families in each sanitary region according to patient’s residence as notified to the National TB Control Program

In **Paraguay**, 110 isolates from the 2005 to 2007 cohort yielded spoligotyping results. The observed frequency was: 37.8% LAM (*n* = 42), 15.5% T (*n* = 17), 15.5% H (*n* = 17), 10.1% S (*n* = 11), 10.1% unknown (*n* = 11), 8.2% new (*n* = 9), 2.8% X3 (*n* = 3) based (Fig. 1, Fig. 2). This distribution follows a similar pattern to that already published by Candia et al., [18] for the strains from the first surveillance cohort. The slight changes are due mainly to reclassification of some patterns of SpolDB4 of the U and T family in SITVITWEB. Among the isolates that could not be classified by SITVITWEB (Additional file 9: Table S1), most belonged to the LAM family according to SpotClust. The distribution within the LAM family was the following: 57.1% LAM9 (*n* = 24), 16.6% LAM4 (*n* = 7), 11.9% LAM3 (*n* = 5), 9.5% LAM6 (*n* = 4), 2.4% LAM5 (*n* = 1), 2.4% LAM (*n* = 1). In terms of SIT distribution, 11.6% SIT42-LAM9 (*n* = 13), 8% SIT34-S (*n* = 9) and 5% SIT391-LAM4 (*n* = 6) where the most frequent ones, as reported before. Genotypes such as SIT2643 (H), SIT2654 (LAM) and SIT2655 (T1) created for orphan strains in the first report of Paraguay [18] were also detected in the 2005–2007 cohort (Additional file 9: Table S2). SIT2643-MIT182 was initially described by Candia et al. [18] and was named “**Tacumbu**” after the men’s correctional facility in Asunción where it was most frequently found. In the 2005–2007 cohort, SIT 2643 was detected in patients residing in Asunción (Additional file 9: Table S2) with a MIRU pattern similar to MIT182.

In Fig. 2 the distribution of the most common spoligotyping family is represented according to the patients’ residence. In the Chaco region (Regions XV, XVI, XVII) with low demographic density, the most common families were LAM and T. In the oriental region (Regions I to XIV and Capital) the clades LAM and T, but also the H and S were ubiquitous. Asunción had the highest notification rate and at least one strain of each of the following genotypes LAM, H, S, T, X, unknown or new was observed.

In **Buenos Aires**, 55 isolates presented results by spoligotyping and frequency was similar to that of Paraguay (Fig. 1), being 40.0% LAM (*n* = 22), 38.2% T (*n* = 21), 14.5% H (*n* = 8), 3.6% S (*n* = 2), 1.8% unknown (*n* = 1) and 1.8% X (*n* = 1). The strain with unknown classification in SITVITWEB displayed the SIT106 genotype. The most frequent SITs were SIT42 (LAM9) and SIT50 (H3). Within the LAM family, the LAM3 genotype was the most frequent (45.5%), besides LAM9 (41.0%), LAM5 (9.0%) and LAM2 (4.5%).

In **Caracas**, amongst the 100 isolates, 64.0% were LAM, 1% was an AG85C SNP positive RD^Rio^ strain that lacked spoligo result and 19.0% were T. Other circulating genotypes were: 7.0% new, 3.0% H, 3.0% unknown SIT106 or SIT4, 1.0% Beijing, 1.0% Manu, and 1.0% ambiguous T3 T2 X1 (Fig. 2). Frequencies within the LAM Family were: 33.8% LAM2 (*n* = 22), 27.7% LAM9 (*n* = 18), 24.6% LAM5 (*n* = 16), 4.6% LAM3 (*n* = 3), 3.0% LAM1 (*n* = 2), 3.0% LAM (*n* = 2) and 1.5% LAM12_MAD1 (*n* = 1). The most prevalent LAM SITs were SIT17 (LAM2) and SIT93 (LAM5), while SIT20, SIT33, SIT42, SIT162, SIT209, SIT376, SIT397, SIT822, SIT1355 and SIT1505 were also detected. According to SpotClust five of the new spoligo types classified as LAM (Additional file 9: Table S1).

### Ag85C SNP as a LAM marker

Considering that there were no profound changes in the bacterial population structure of **Paraguay** as defined by spoligotyping from 2003 to 2007, we further concentrated on isolates of the LAM family by determining the presence of the LAM co-marker, the SNP Ag85C(G-A) (Table 1).

**Table 1:** Spoligotyping versus Ag85C (G-A) SNP for LAM detection

		Spoligotyping		
	^a^AG85C	LAM	Not LAM	New, orphan, unknown
PARAGUAY	SNP	105	9	19
	No SNP	4	97	14
	Mixed	2	7	1
ARGENTINA	SNP	22	13	1
	No SNP	0	18	0
	Mixed	0	1	0
VENEZUELA	SNP	63	0	7
	No SNP	0	23	4
	Mixed	0	2	1

^a^Number of isolates with presence or absence of the Ag85C^103^ SNP. Mixed = two amplification products indicating presence and absence of the SNP. In this table strains that lacked result for spoligotyping or the SNP were excluded

**Fig. 3:**
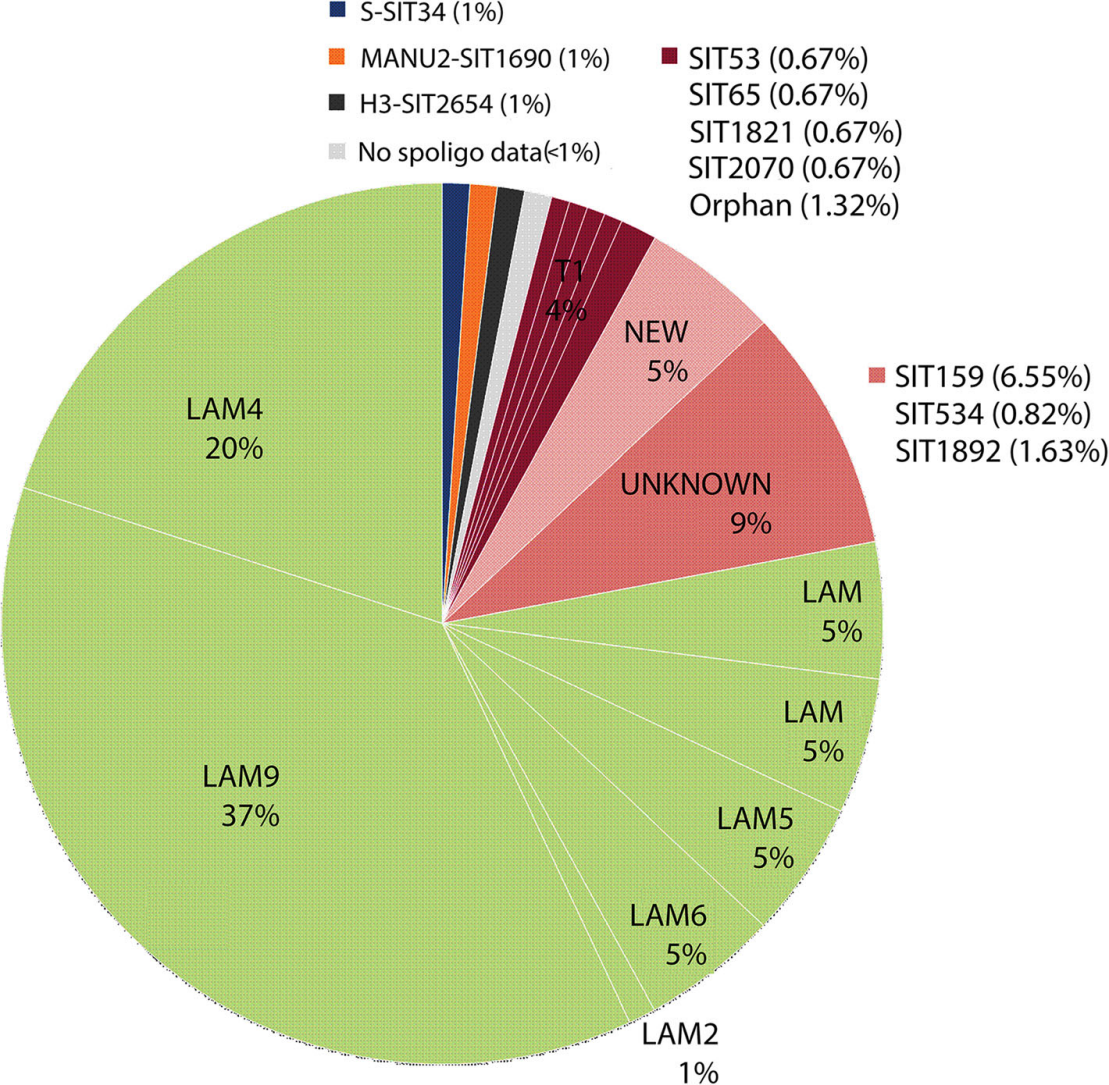
Distribution of the spoligotyping families of the SNP Ag85C positive strains among Paraguayan isolates

**Fig. 4:**
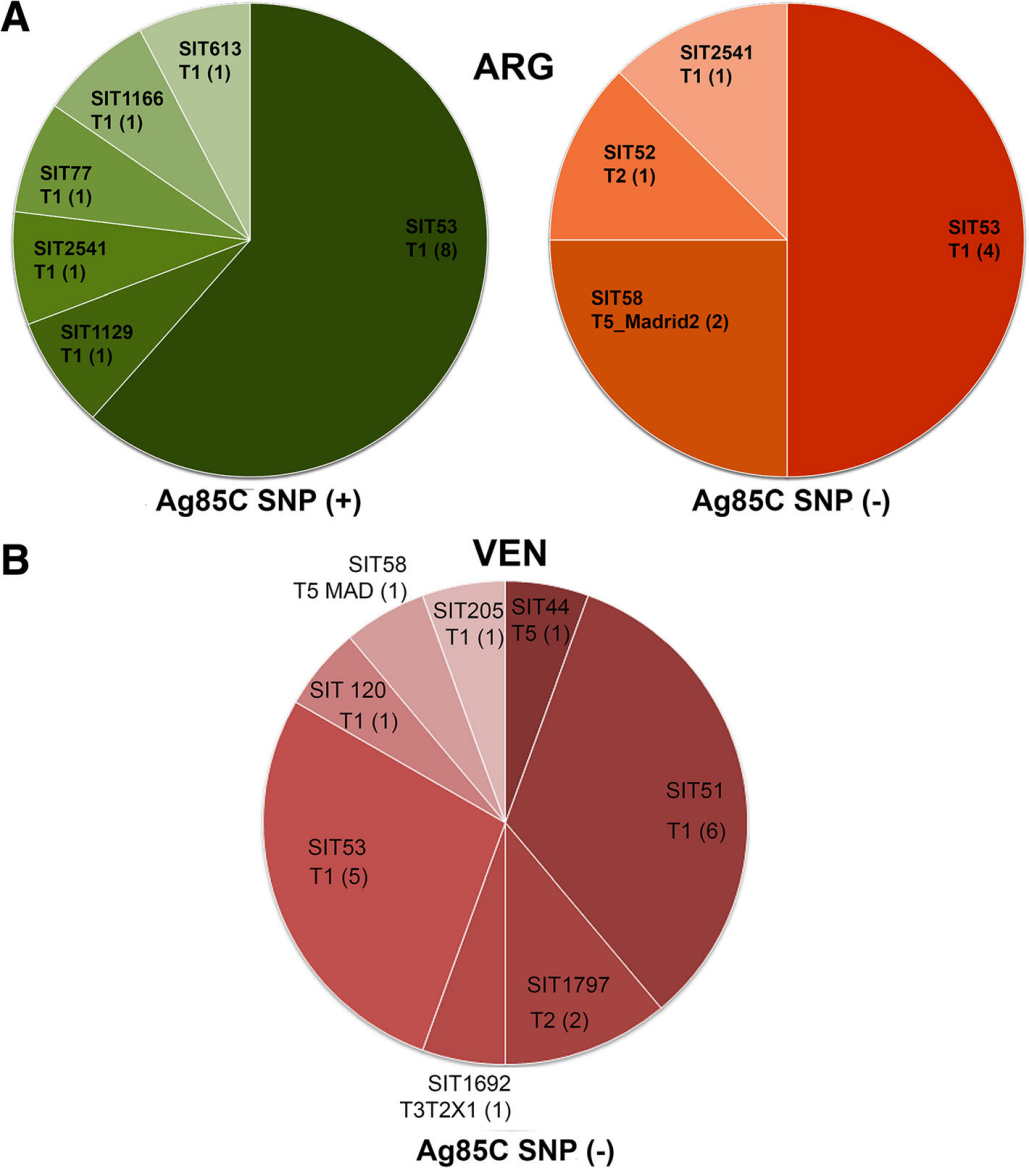
Distribution of the spoligotyping families of the SNP Ag85C positive or negative strains from **a** Buenos Aires- Argentina **b** Caracas- Venezuela

Amongst the strains from both cohorts of Paraguay, a total of 50.6% (133 of 263) presented the Ag85C SNP, 38.5% did not; 3.5% had mixed results and 1.1% did not yield amplification products. Among the 133 SNP positive strains, 78% were LAM, 14% were new or had unknown patterns, 7% were non-LAM genotypes (Fig. 3). We cross referenced the non-LAM Ag85C SNP positive strains with MIRU or *IS6110* RFLP results (Additional file 1: Figure S1). A total of 26 out of 28 non- LAM SNP positive strains could be linked to LAM strains by one or both techniques (Additional file 9: Table S3). Amongst the SNP negative strains, 84.3% were non-LAM, 12.2% had new or unknown patterns and 3.5% were LAM. Within the Ag85C SNP negative LAM strains we detected two strains with SIT125 (LAM3). In samples from **Buenos Aires**, the Ag85C SNP was present in all LAM strains, in 13 of the 21 T strains (Fig. 4a) and in one SIT106 strain. Strains from the other families, like H, X, S and also 8 of 21 T strains (Fig. 4a), did not present the SNP. In the samples from **Venezuela,** the Ag85C SNP was present in all the LAM strains as well as in a SIT106 strain and six orphan strains (Fig. 4b, Additional file 9: Table S1). Overall for the three populations a positive significant association was observed for the presence of the SNP in the “LAM” versus non-LAM isolates (*p* < 0.05, Chi SquareTest, α = 0.05). In the Ag85C SNP negative group, strains belonged to T, H or X family, new (T1 by Spotclust) and unknown (SIT4).

### Detection of RD^Rio^ and RD174

In this study we also screened for the presence of the RD^Rio^ and RD174 LSP as described by Gibson et al., [24]. For **Paraguay** we detected a 10% prevalence among the 254 isolates with results for the LSP, corresponding to 27 isolates from 26 patients. Furthermore, one isolate (PY394) lacked RD^Rio^ deletion but presented an intact RD174. In addition, nine isolates presented a mixed result for the RD^Rio^ LSP. The RD^Rio^ strains belonged to the LAM9, LAM5, LAM4, LAM2, LAM1 subtypes or a new pattern and essentially displayed the MIRU02-MIRU40 signature. The RD^Rio^ lineage was distributed in most Sanitary Regions. (Additional file 9: Table S4). The 12 loci MIRU based “Minimum Spanning Tree” (MST) (Additional file 2: Figure S2) for the LAM strains showed a central node (PY307) with a MIRU pattern described as the hypothetical founder of the RD^Rio^ family (MIRU 224226153321).

Among the strains that did not present the RD^Rio^ LSP, 99.6% of them did not present the RD174 deletion either. Noteworthy one particular strain (PY316) presented only a faint PCR band for RD^Rio^ and the RD174 region was deleted. In the MIRU based MST this strain was located in the interface between RD^Rio^ negative strains and RD^Rio^ positive strains.

As far as the isolates from **Buenos Aires**, a total of six strains (11.3%) presented the RD^Rio^ deletion along with other features for the lineage. They belonged to LAM9-SIT42 and LAM5-orphan. Three strains presented a mixed profile for RD^Rio^ (Additional file 9: Table S4). In the MIRU based MST (Additional file 3: Figure S3) we can observe a grouping containing RD^Rio^ strains and the hypothetical founder ARG015.

In **Venezuela,** a total of 47 strains presented the RD^Rio^ deletion along with the other features of the lineage. These strains belonged to LAM9-SIT42, LAM2-SIT17, LAM9-SIT1505 and LAM5-SIT93, LAM1-SIT20, LAM9-SIT162 and a new genotype. Six strains presented a mixed profile for the RD^Rio^ deletion. One strain (VEN1362) presented a mixed profile for both RD^Rio^ and RD174. The MIRU based MST (Additional file 4: Figure S4) showed a node containing a LAM9 isolate (VEN1420) with the hypothetical founder pattern. Within this node a sample (VEN1339) with mixed RD^Rio^ pattern and LAM5 genotype was also found. The MST separated RD^Rio^ and non RD^Rio^ strains in different groups.

Combining the results of the **three countries** (*n* = 399; Table 2), the relation between the detection of RD^Rio^ and RD174 showed a significant positive association of 99.86%. Amongst the strains with the RD174 deletion, 96.15% had the RD^Rio^ deletion. And amongst the strains with the RD^Rio^ deletion, 93.75% had the RD174 deletion.

**Table 2:** Detection of RD^Rio^ versus RD174 in isolates from Paraguay, Buenos Aires and Caracas

	RD^Rio^	Non RD^Rio^	Mixed
Deleted RD174	**75**	**3**	3
Not Deleted RD174	**5**	**303**	3
Mixed	3	2	2

### MIRU-VNTR typing

We performed MIRU-VNTR typing on LAM strains and Ag85C SNP positive strains while only some non-LAM strains were included as outgroups. The computer-generated UPGMA MIRU dendogram for isolates from **Paraguay** essentially showed a grouping of LAM and another of non-LAM genotypes with T isolates distributed amongst both. Combined MIRU and spoligotyping showed the existence of 25 clusters containing 65 isolates and two to six strains per cluster (Additional file 5: Figure S5). Amongst the Ag85C SNP positive strains, 16 clusters with 40 isolates having two to four strains per cluster could be detected. Results were compared to available *IS6110* RFLP data to confirm clustering. For example, a SIT93-LAM5 cluster was detected by both. This genotype circulated mostly in the Chaco, Sanitary region XVII (Additional file 6: Figure S6). In Sanitary Regions XI and XIV, four SIT391-LAM4 clusters were also detected by both methods. This SIT391 has been reported in Brazil (*n* = 2) and Paraguay (*n* = 27) according to SITVITWEB and our results. MIRU analysis also revealed several SIT42-LAM9 clusters that were in agreement with *IS6110* RFLP clusters. For example, a SIT42-LAM9 cluster containing strains obtained from male inmates (PY127 and PY157). Also, three SIT42-LAM9 clusters containing RD^Rio^ strains. Other *IS6110* RFLP clusters [18], like those harboring SIT177-LAM9 or SIT2647-LAM9 strains respectively were also confirmed. Furthermore, MIRU detected a new cluster harboring SIT1610-LAM6 strains. This genotype was previously detected in one isolate (PY237) in 2003 [18]. Four more strains were detected afterwards and they were all closely related. A SIT2654 cluster (PY170, PY104, PY203) was detected by *IS6110* RFLP in samples from 2002 to 2003 [18]. An additional SIT2654 genotype (PY388) was detected in 2007. The close association between SIT2654 strains was confirmed by MIRU. Two SIT2654 strains (PY170, PY388) were isolated in the capital of the Sanitary Region VII (Additional file 6: Figure S6). PY104, PY203 were resistant to rifampicin and isolated from the same patient in 11/ 2002 and 07/ 2003.

Although MIRU 12 has shown several concordant results with *IS6110* RFLP, it also showed limitation in its discriminatory power when it came to similar genotypes within the LAM family like clusters containing SIT391-SIT93, SIT753-SIT42, SIT1367-SIT828-SIT42 or SIT93-SIT578 respectively (Additional file 5: Figure S5).

The MIRU based dendogram for the strains from **Buenos Aires** showed three clusters with two strains each (Additional file 7: Figure S7), i.e., a RD^Rio^ cluster as well as a SIT53-T1 and a MIT816 cluster. The dendogram also showed that Ag85C SNP positive T strains were mostly associated to LAM. Accordingly, the T strains without the SNP were associated to non-LAM genotypes.

The MIRU results for strains from **Caracas** showed eight clusters containing 43 isolates (Additional file 8: Figure S8). Five of the eight clusters harbored LAM strains. The largest cluster contained 12 RD^Rio^ strains with 11 SIT17-LAM2 genotypes and one SIT93-LAM5 genotype. Another all RD^Rio^ cluster contained two SIT42, eight SIT93 and one orphan spoligo pattern (777357607760771) that classified as LAM9 by Spotclust. Another cluster (VEN1330, VEN1334) harbored SIT42-LAM9 strains which presented the RD^Rio^ deletion, but neither presented the deletion in RD174. In the sample population from Caracas, the 12 loci MIRU approach showed limitation in differentiating similar genotypes like SIT42, SIT17 and SIT93, all members of the LAM family.

The allelic diversity (h) for each minisatellite [36] is shown in Additional file 9: Table S5 (A-C), for Ag85C SNP positive strains from **Paraguay, Buenos Aires and Caracas** respectively. The allelic diversity of Ag85C SNP positive strains from the **three countries** was also compared to the allelic diversity within the LAM family published by Demay et al. [11] (Additional file 9: Table S6). Overall the most polymorphic loci in this study were MIRU40, MIRU31, MIRU10, MIRU26, MIRU16. The least polymorphic were: MIRU24, MIRU20, MIRU04, MIRU23.

The relation between MIRU signature and the RD^Rio^ deletion was also analyzed for the combined results from the three countries (Additional file 9: Table S7). We observed that 97.5% of the RD^Rio^ isolates presented two allelic copies for the MIRU02 locus and 96% presented 1 allelic copy for the MIRU40 locus.

**Fig. 5:**
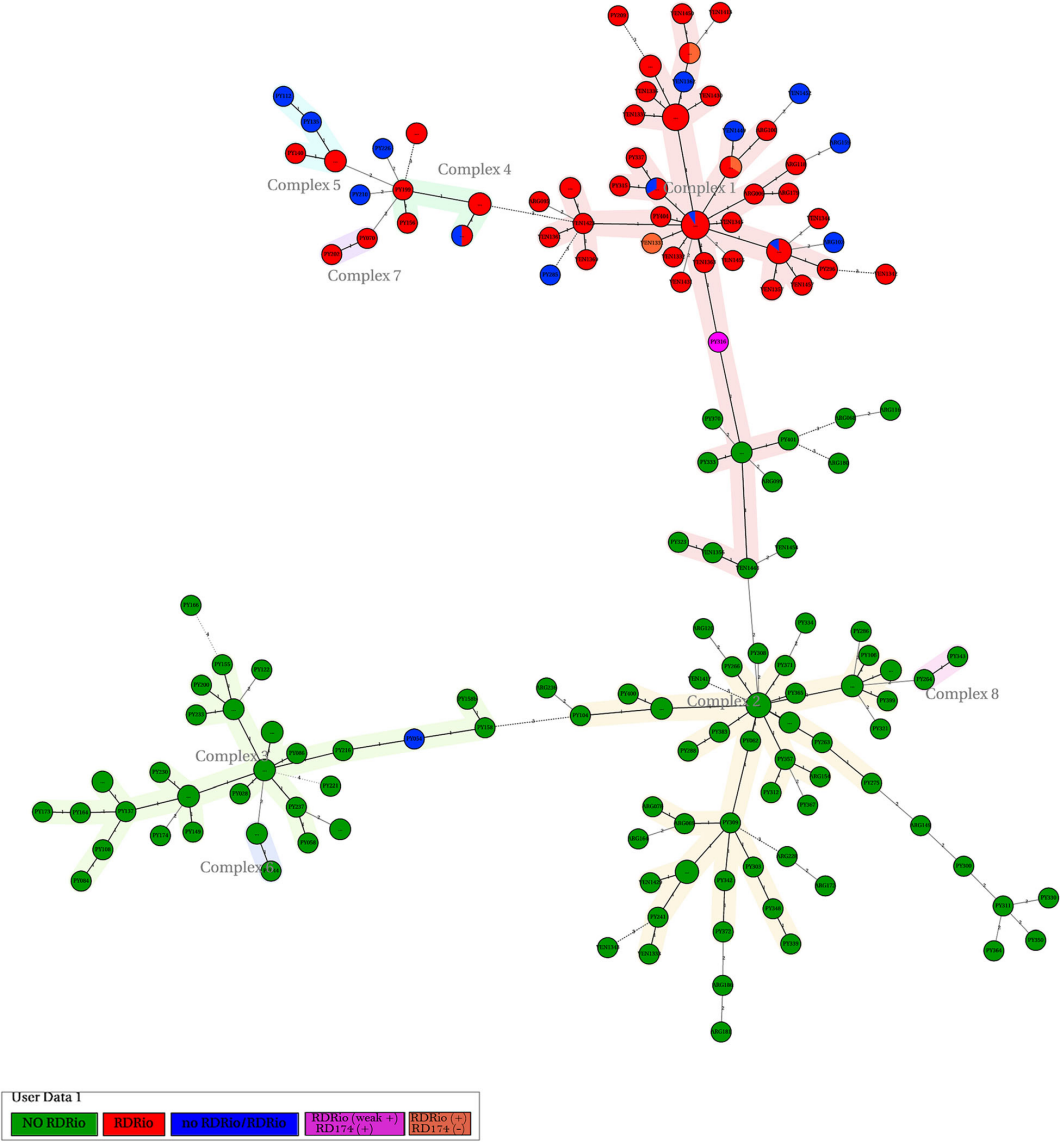
Minimum spanning Tree based on 12 loci MIRU-VNTR profiles of Ag85C SNP positive strains from Buenos Aires, Caracas and Paraguay. Each node represents a MIRU-VNTR type. The size of the circle is relative to the number of isolates with the MIRU pattern and the colors indicate clusters containing either RD^Rio^ (red), WT (green) genotypes or mixed pattern (blue). In orange are strains with RD^Rio^ deletion but no RD174 deletion. The theoretical progenitor MIRU-VNTR-type for RD^Rio^ (MIRU 224226153321) is present in complex 1

The overall MIRU based MST of LAM or Ag85C SNP positive strains from **Paraguay, Buenos Aires** and **Caracas** (Fig. 5), displays the agglomeration of RD^Rio^ strains,presenting a central node with the hypothetical founder MIRU pattern (PY307, ARG015, VEN1420, VEN1339). PY316 with the RD174 deletion and a dubious RD^Rio^ result was the connecting node with the non RD^Rio^ strains.

## Discussion

Molecular genetic studies of circulating Mtb strains allows monitoring of strain dispersal and evolutionary adaptations, important for better understanding of bacterial and disease dissemination. Herein we presented the molecular patterns of LAM isolates from different locations in South-America. An initial approach using spoligotyping confirmed the well-known importance of the LAM family regionally. In **Paraguay** the LAM (37.8%) and T (15.5%) genotypes constituted about 50.0% of the circulating genotypes during 2002–2007. Nationwide the LAM family was the most ubiquitous and the circulating sub-lineages were LAM9, LAM4, LAM3, LAM6, LAM5, LAM in decreasing order of prevalence. In the sample population of **Buenos Aires**, the most prevalent genotypes belonged to the LAM (40.0%) and T (38.2%) clades. Circulating LAM lineages were LAM3, LAM9, LAM5 and LAM2 in decreasing order of prevalence. The results are concordant with previous reports [15, 37]. Amongst the samples from **Caracas,** LAM (65.0%) and T (20.0%) were the most prevalent clades. In this population LAM2-SIT17 (22.4%) and LAM5-SIT93 (16.3%) were the most frequent genotypes. Previous reports for **Venezuela** [38] showed that during 1996–2006, that SIT17 and SIT93 represented the most frequent spoligotype (20.0 and 8.7% respectively) found in **Caracas**.

It is common knowledge that mainly because of homoplasy, spoligotyping has limited resolution power and that discrepancies can be obtained when comparing spoligotyping and other approaches such as MIRU-VNTR typing, IS6110-RFLP and characterization of SNPs and LSPs [22, 36, 39, 40]. So, in this study, we screened the LAM family combining different techniques to achieve a better resolution. We started by evaluating the Ag85C SNP as a marker for the LAM lineage and detected a positive correlation of almost 100% in the three countries. In some cases, non-LAM strains also presented the SNP. If we consider that amongst the **Paraguayan** strains, 25 out of 28 non-LAM strains with the SNP were closely related to the LAM clade by RFLP or MIRU, the overall prevalence of LAM strains would rise to about 50%. Under this rationale, in the sample population of **Buenos Aires,** it would increase to about 60% and in **Caracas** to 71%.

In the first report for Paraguay and based on *IS6110* RFLP results, Candia et al. [18] had proposed new SITs (SIT2646, SIT2651, SIT2652, SIT2648, SIT2647, SIT2650, SIT265 and SIT2649) to represent genotypes that were linked to the LAM family, but at that time had no classification. We checked the Ag85C SNP and they all turned out to be positive.

Although Whole Genome Sequencing (WGS) for Mtb has a great potential as a diagnostic, epidemiologic, and research tool, caution is still needed when interpreting WGS data as there are some important limitations; from correct interpretation of drug susceptibilities to the bioinformatic support needed [41]. In fact, genotyping by MIRU-VNTR 24 loci is currently considered the reference method [42]. But to better understand the practical utility and added value of increased discriminatory power associated with the additional 12 loci in genotyping and TB transmission, it must be verified by epidemiologic investigations in different settings [43] . Several studies have demonstrated variability in the level of polymorphism of the MIRU-VNTR loci that can be attributed to the geographic origin of the strain and to differences between genetic lineages [22, 44, 45]. Furthermore, based on the allelic diversity, there is previous evidence suggesting that the number and combination of loci to be used to discriminate between genotypes can be adapted to the population of Mtb strains [46–48]. In this study, applying 12 loci MIRU [49, 50] allowed us to detect the existence of clusters with members of the LAM, H or S families respectively, with the presence of T isolates inserted in between. Interestingly, when evaluating conflicting genotypes, Ag85C SNP and Spotclust classification showed to be useful to solve the issues. Examples of these cases were the grouping of Ag85C SNP positive isolates of the T or U family with members of the LAM family, or the grouping of LAM3-S isolates (all Ag85C SNP negative) with members of the S family. The SNP positive T strains that were closely related to LAM strains based on MIRU (> 96% similarity) showed different SITs like SIT163, SIT53, SIT1129, SIT77 SIT1166. Also, in our study, SIT159 (Unknown) strains that used to be T1 Tuscany in SpolDB4 all presented the AG85C SNP and related closely to LAM strains by RFLP or MIRU. This was reported before for this genotype [2, 51, 52]. We also observed that genotypes with SIT397, SIT1241, SIT1758 or SIT1892, considered as U patterns in SpolDB4 and that were reclassified as LAM by SITVITWEB, all presented the Ag85C SNP and even classified as LAM by Spotclust. Absence of the Ag85C SNP in LAM isolates was detected in two LAM3 SIT125 isolates from **Paraguay**. SIT125 used to T2 family in spolDB4. In the MIRU based dendogram they grouped with SIT4 strains that did not present the Ag85C SNP. We also observed that no SIT4 (LAM3-S convergent in SpolDB4, ¨unknown¨ in SITVITWEB) genotype presented the Ag85C SNP. This is in concordance with results based on SNP lineage assignation [51]. Most strains from the other families like H, X, S did not present the SNP. The SNP negative T strains associated to non-LAM families by MIRU showed different SITs like SIT53, SIT58, SIT52, SIT2541. Finally, the few non-LAM isolates (X, H or T) that did present the Ag85C SNP were highly similar to LAM isolates according to MIRU or RFLP. Overall, the combination of MIRU 12 with the Ag85C SNP has shown to be useful in discriminating conflicting spoligotypes.

Within the LAM family, the limitation of the MIRU 12 loci technique could be observed in some cases, like clusters containing LAM3 isolates with SIT33 and SIT130 or with SIT42, SIT93, or SIT93 that only differ in one spacer.

In terms of discriminating power within the LAM family, we concluded that MIRU40, MIRU31, MIRU26, MIRU16 and MIRU10 were highly discriminatory and MIRU24, MIRU23, MIRU20 and MIRU4 were the least discriminatory. Nevertheless, we should keep in mind that in settings with high RD^Rio^ (or LAM2) prevalence like **Venezuela**, MIRU40 loses its discriminatory power. In fact, for the sample population of Venezuela MIRU26 had the highest discriminatory power. In terms of the least discriminatory loci, **the three locations** presented MIRU04, MIRU20 and MIRU23 as most common. Furthermore, we observed a MIRU signature in most SNP positive strains, i.e., two allelic copies in MIRU04, six copies in MIRU23, and two copies in MIRU20. Another frequent pattern in LAM strains was the presence of one allelic copy in MIRU24 and two copies in MIRU02, though some AG85C SNP positive T strains did not show this.

The other focus of this study was the evaluation of the RD^Rio^ lineage. Firstly, we confirmed the importance of RD174 as an RD^Rio^ lineage co-marker, detecting a ≥ 99.86% association rate. When being screened for their frequency, only 10% RD^Rio^ were observed among isolates from Paraguay. In **Rio de Janeiro**, initially a 30% (mostly LAM9 and LAM2) prevalence was reported amongst 336 patients diagnosed between 2002 and 2003 [25]. Later on, an 11% frequency of RD^Rio^ (mostly LAM9 and LAM2) was reported amongst 186 patients diagnosed between 2008 and 2009 [30]. In **Rio Grande do Sul**, the southernmost Brazilian state, a frequency of 28.9% was detected amongst 45 clinical isolates obtained between 1998 and 2001 [53]. In the same state, amongst 237 isolates collected between 2004 and 2006, a total of 38% was RD^Rio^ [29]. In **Espírito Santo**, southeast Brazil, amongst 981 strains collected during 2000–2010, 38% was detected to be RD^Rio^ [54]. In **Portugal**, amongst 859 isolates, one third was RD^Rio^, most of them LAM1 and MDR [28, 55]. LAM1 and LAM2 families are recognized to be exclusives of the RD^Rio^ lineage and LAM3 to be exclusive of LAM-WT [25]. Interestingly, the low proportion of RD^Rio^ isolates in **Paraguay**, was accompanied by a low proportion of LAM1 and LAM2 isolates. The LAM3 strains were exclusively of the LAM-WT genotype. The LAM4 and LAM5 families showed the two genotypes, and LAM6, LAM10 and LAM9-S harbored exclusively LAM-WT isolates. Amongst the **Argentinean** strains only 11% were RD^Rio^ and this low proportion might be explained by the high frequency of LAM3 strains found in the sample population. All the **Argentinean** RD^Rio^ isolates belonged to LAM2, LAM5 and LAM9 genotypes and the proportion of LAM9-WT vs LAM9-RD^Rio^ was equal. Amongst the **Venezuelan** strains a remarkably high frequency (55%) of the RD^Rio^ type was detected. This result is in concordance with the predominance of LAM2 isolates reported here and in an earlier study [38]. All of the Venezuelan LAM1 and LAM2 isolates belonged to the RD^Rio^ lineage and again, no RD^Rio^ isolate belonged to the LAM3 family. The MIRU-VNTR based MST of each country displayed a node with a LAM9-RD^Rio^ hypothetical founder strain. Overall, amongst the RD^Rio^ isolates, MIRU26 was highly polymorphic, while MIRU27, MIRU10, MIRU31, MIRU39, MIRU16 were moderately polymorphic. The other loci almost lacked polymorphism and in particular, respectively 97.5 and 96% of the RD^Rio^ isolates presented two allelic copies in MIRU02 and one in MIRU40. To underline the importance of this marker, we need to remember that MIRU40 is considered a highly polymorphic locus for LAM strains [13, 56–59].

In the present study, the LAM population structure varied considerably according to the geographical location. Differential adaptation of LAM strains is under continuous analysis [60]. The difference of success of the RD^Rio^ lineage might be related to genetic, cultural or environmental characteristics of the host population. Advanced techniques are necessary to further understand the behavior of LAM in neighboring populations like Paraguay, Argentina, Brazil and Venezuela.

## Conclusion

We confirmed the importance of different circulating LAM sub-types in the TB epidemic in South America. Submitting LAM strains to genotyping by MIRU-VNTR and co-markers like the SNP Ag85C103 (G-A), RD174, RD^Rio^ certified their usefulness within workflows to detect these strains that are endemic and highly prevalent regionally.

## Additional files

Additional file 1: **Figure S1.** Computer-generated dendrogram according to UPGMA IS6110-RFLP analysis of selected strains from Paraguay using Bionumerics v4.50 (Applied Maths). Spoligotyping results are also displayed. (TIF 716 kb)

Additional file 2: **Figure S2.** Minimum spanning Tree based on 12 loci MIRU-VNTR profiles of Ag85C SNP positive strains from Paraguay. Each node represents a MIRU-VNTR type. The size of the circle is relative to the number of isolates with the MIRU pattern and the colors indicate clusters containing either RD^Rio^ (red), WT (green) genotypes or mixed pattern (blue). In orange are strains with RD^Rio^ deletion but no RD174 deletion. (TIF 759 kb)

Additional file 3: **Figure S3.** Minimum spanning Tree based on 12 loci MIRU-VNTR profiles of the strains from Buenos Aires, Argentina. Each node represents a MIRU-VNTR type. The size of the circle is relative to the number of isolates with the MIRU pattern and the colors indicate clusters containing either RD^Rio^ (red), WT (green) genotypes or mixed pattern (blue). (TIF 352 kb)

Additional file 4: **Figure S4.** Minimum spanning Tree based on 12 loci MIRU-VNTR profiles of Ag85C SNP positive strains from Caracas, Venezuela. Each node represents a MIRU-VNTR type. The size of the circle is relative to the number of isolates with the MIRU pattern and the colors indicate clusters containing either RD^Rio^ (red), WT (green) genotypes or mixed pattern (blue). (TIF 342 kb)

Additional file 5: **Figure S5.** Computer-generated UPGMA dendrogram based on combined 12 loci MIRU-spoligotyping analysis of selected strains from Paraguay, From left to right *i)* Boxes colors: in red RD^Rio^ strains, in green no-RD^Rio^ strains, in blue mixed RD^Rio^/no-RD^Rio^, PY316 colored in purple indicating a seemingly weak positive result for RD^Rio^ deletion and positive for RD174 deletion. Boxes identification: SNP or NO SNP refers to Ag85C SNP positive or negative strains respectively, *ii)* strain identification, *iii)* SITVITWEB classification *iv)* SIT form SITVITWEB, *v)* SIT from SpolDB4, *vi)* SpolDB4 classification, *vii)* 12 loci MIRU profile (MIRU02, MIRU04, MIRU40, MIRU10, MIRU16, MIRU20, MIRU23, MIRU24, MIRU26, MIRU27, MIRU31, MIRU39, MIRU40), *viii)* spoligotyping profile. (TIF 1164 kb)

Additional file 6: **Figure S6.** Distribution of the LAM sub-families in each sanitary region according to patient’s residence as notified to the National TB Control Program. (TIF 1222 kb)

Additional file 7: **Figure S7.** Computer-generated UPGMA dendrogram based on combined 12 loci MIRU-spoligotyping analysis of selected strains from Buenos Aires-Argentina, From left to right *i)* Boxes colors: in red RD^Rio^ strains, in green no-RD^Rio^ strains, in blue mixed RD^Rio^/no-RD^Rio^, Boxes identification: SNP or NO SNP refers to Ag85C SNP positive or negative strains respectively, *ii)* strain identification, *iii)* SITVITWEB classification *iv)* SIT form SITVITWEB, *v)* SIT from SpolDB4, *vi)* SpolDB4 classification, *vii)* 12 loci MIRU profile (MIRU02, MIRU04, MIRU40, MIRU10, MIRU16, MIRU20, MIRU23, MIRU24, MIRU26, MIRU27, MIRU31, MIRU39, MIRU40), *viii)* spoligotyping profile. (TIF 422 kb)

Additional file 8: **Figure S8.** Computer-generated UPGMA dendrogram based on combined 12 loci MIRU-spoligotyping analysis of selected strains from Caracas-Venezuela, From left to right *i)* Boxes colors: in red RD^Rio^ strains, in green no-RD^Rio^ strains, in blue mixed RD^Rio^/no-RD^Rio^, Boxes identification: SNP or NO SNP refers to Ag85C SNP positive or negative strains respectively, *ii)* strain identification, *iii)* SITVITWEB classification *iv)* SIT form SITVITWEB, *v)* SIT from SpolDB4, *vi)* SpolDB4 classification, *vii)* 12 loci MIRU profile (MIRU02, MIRU04, MIRU40, MIRU10, MIRU16, MIRU20, MIRU23, MIRU24, MIRU26, MIRU27, MIRU31, MIRU39, MIRU40), *viii)* spoligotyping profile. (TIF 1017 kb)

Additional file 9: **Table S1.** Isolates with no classification by SITVITWEB and their corresponding Spotclust results. **Table S2.** Orphan strains detected in Paraguay by Candia et al., (2007) are detected again in the 2005–2007 cohort. **Table S3.** Ag85C SNP positive strains that were not classified as LAM by spoligotyping. **Table S4.** RDRio strains. **Table S5.** Allelic diversity (h) among the LAM and/or Ag85C SNP positive strains from Argentina, Paraguay and Venezuela. **Table S6.** Allelic diversity (h) of MIRU minisatellite loci among the LAM or Ag85C SNP positive strains. **Table S7.** Copy number in MIRU02 and MIRU40 in RDRio strains from Paraguay, Buenos Aires and Caracas. (XLS 71 kb)

## Abbreviations

ARG: Argentina
LAM: Latin American & Mediterranean
LSP: Single nucleotide polymorphismLong sequence polymorphism
MIRU-VNTR: Mycobacterial interspersed repetitive unit-variable number tandem repeats
MSPyBS: Paraguayan Ministry of Health
Mtb: *Mycobacterium tuberculosis*
PY: Paraguay
RD: Region of Difference
RFLP: Restriction Fragment Length Polymorphism
SIT: Standard International types
VEN: Venezuela
WT: Wild type
