# Supplementary figures and images for: Exploring the “Latin American Mediterranean” family and the RDRio lineage in Mycobacterium tuberculosis isolates from Paraguay, Argentina and Venezuela
Source: BMC Microbiol. 2019 Jun 13;19:131. doi: 10.1186/s12866-019-1479-6 (PMC6567603; doi:10.1186/s12866-019-1479-6)

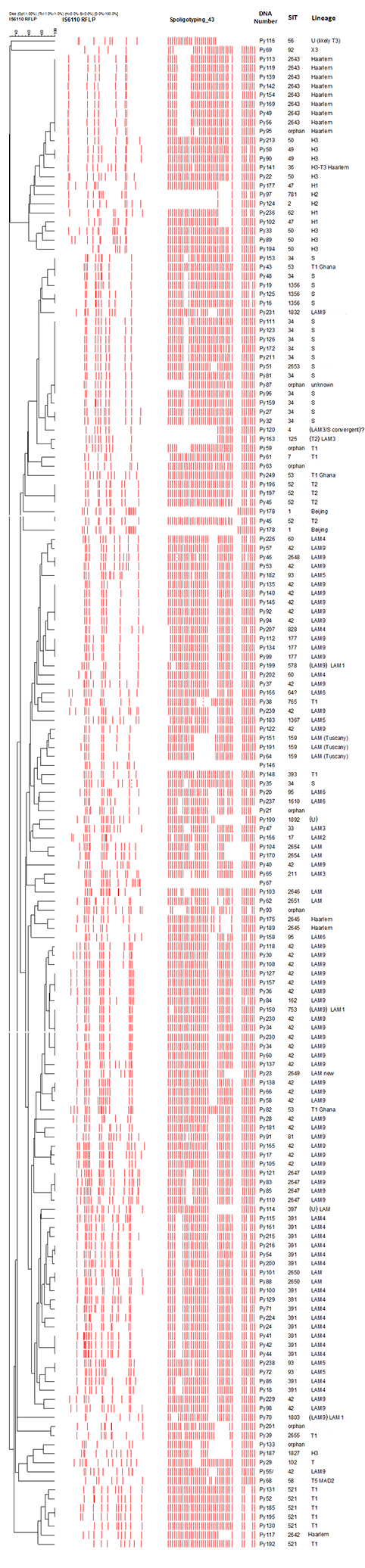

Supplement: Supplementary file 1 — Figure S1. Computer-generated dendrogram according to UPGMA IS6110-RFLP analysis of selected strains from Paraguay using Bionumerics v4.50 (Applied Maths). Spoligotyping results are also displayed. (TIF 716 kb) [file 12866_2019_1479_MOESM1_ESM.tif]

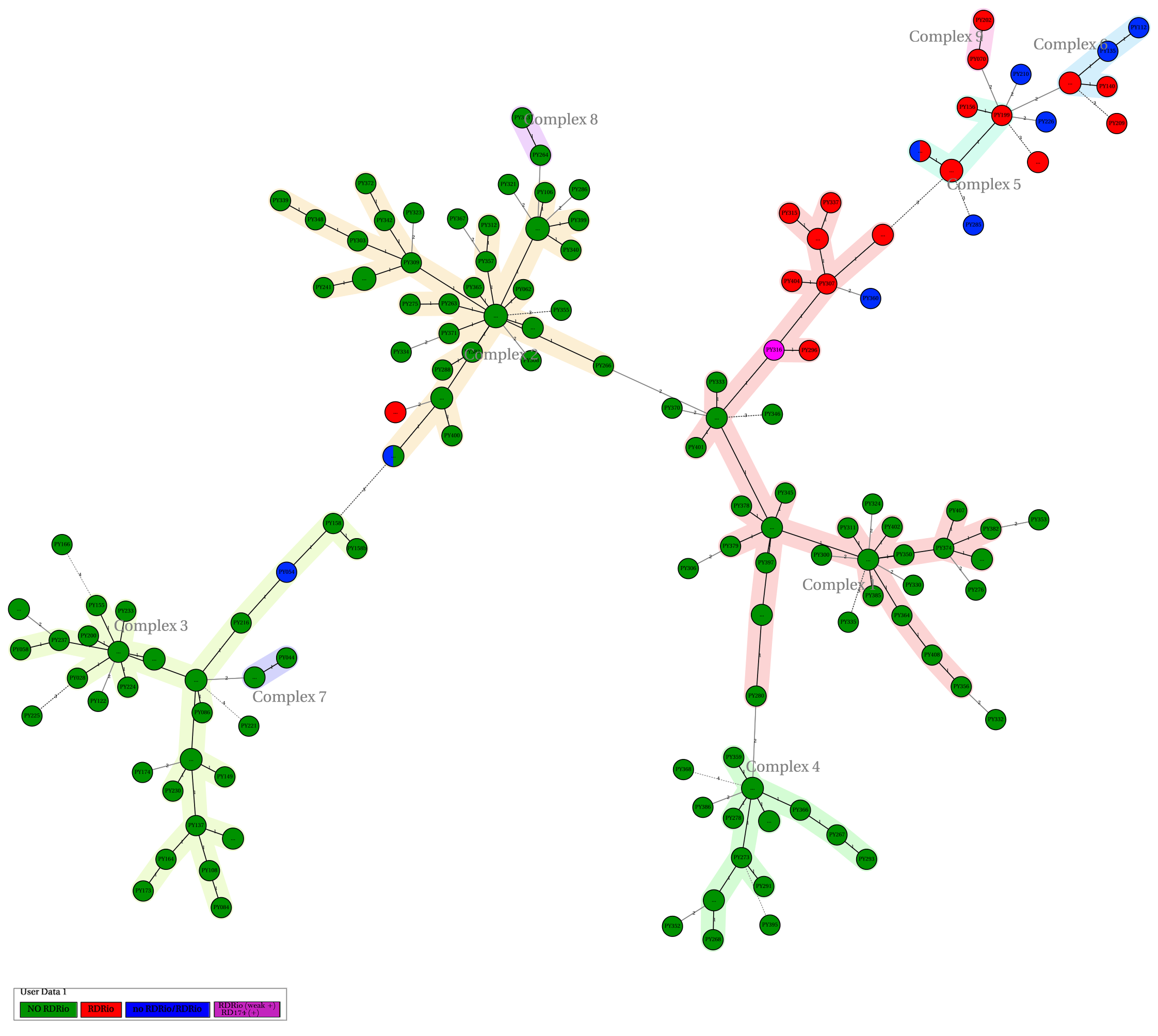

Supplement: Supplementary file 2 — Figure S2. Minimum spanning Tree based on 12 loci MIRU-VNTR profiles of Ag85C SNP positive strains from Paraguay. Each node represents a MIRU-VNTR type. The size of the circle is relative to the number of isolates with the MIRU pattern and the colors indicate clusters containing either RDRio (red), WT (green) genotypes or mixed pattern (blue). In orange are strains with RDRio deletion but no RD174 deletion. (TIF 759 kb) [file 12866_2019_1479_MOESM2_ESM.tif]

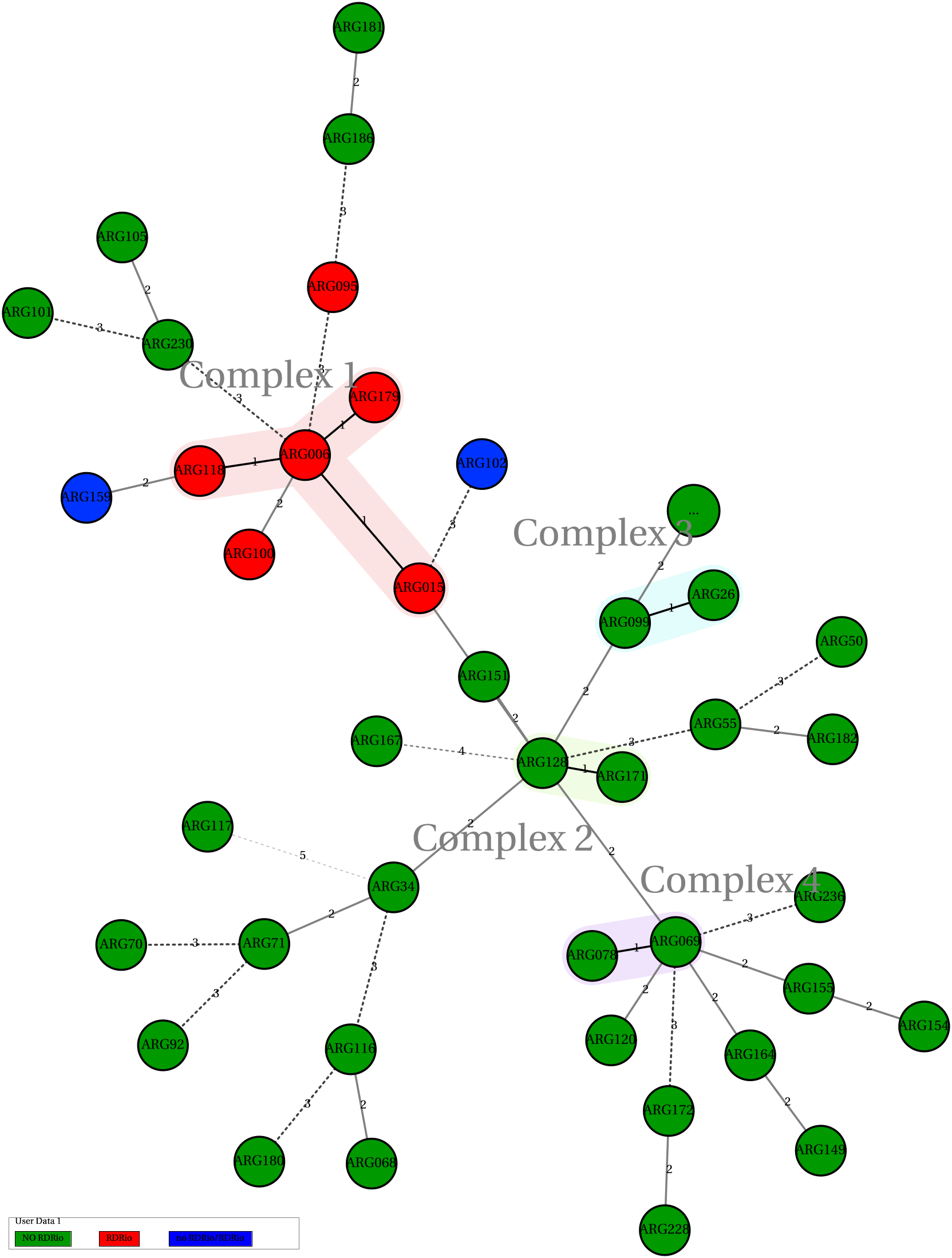

Supplement: Supplementary file 3 — Figure S3. Minimum spanning Tree based on 12 loci MIRU-VNTR profiles of the strains from Buenos Aires, Argentina. Each node represents a MIRU-VNTR type. The size of the circle is relative to the number of isolates with the MIRU pattern and the colors indicate clusters containing either RDRio (red), WT (green) genotypes or mixed pattern (blue). (TIF 352 kb) [file 12866_2019_1479_MOESM3_ESM.tif]

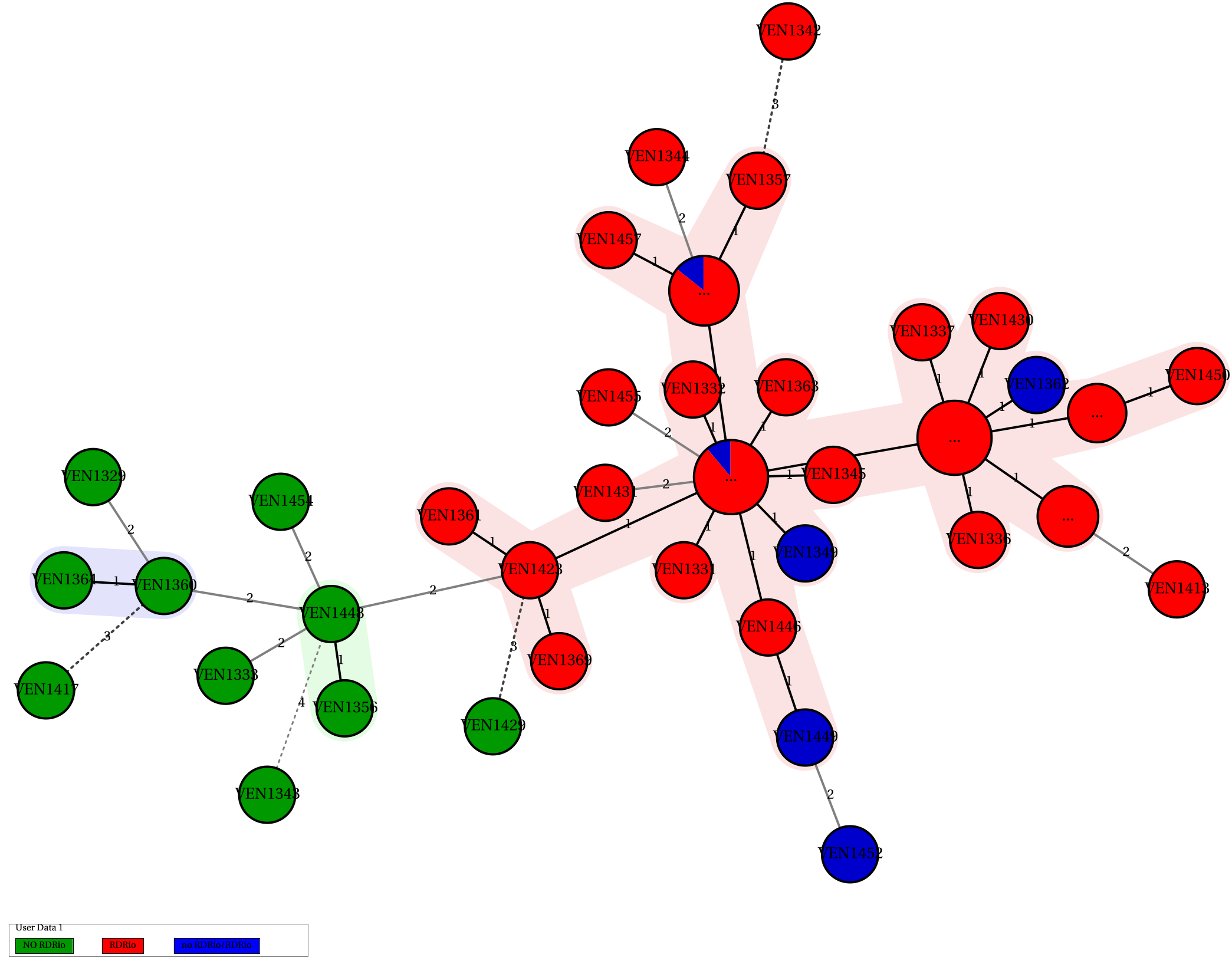

Supplement: Supplementary file 4 — Figure S4. Minimum spanning Tree based on 12 loci MIRU-VNTR profiles of Ag85C SNP positive strains from Caracas, Venezuela. Each node represents a MIRU-VNTR type. The size of the circle is relative to the number of isolates with the MIRU pattern and the colors indicate clusters containing either RDRio (red), WT (green) genotypes or mixed pattern (blue). (TIF 342 kb) [file 12866_2019_1479_MOESM4_ESM.tif]

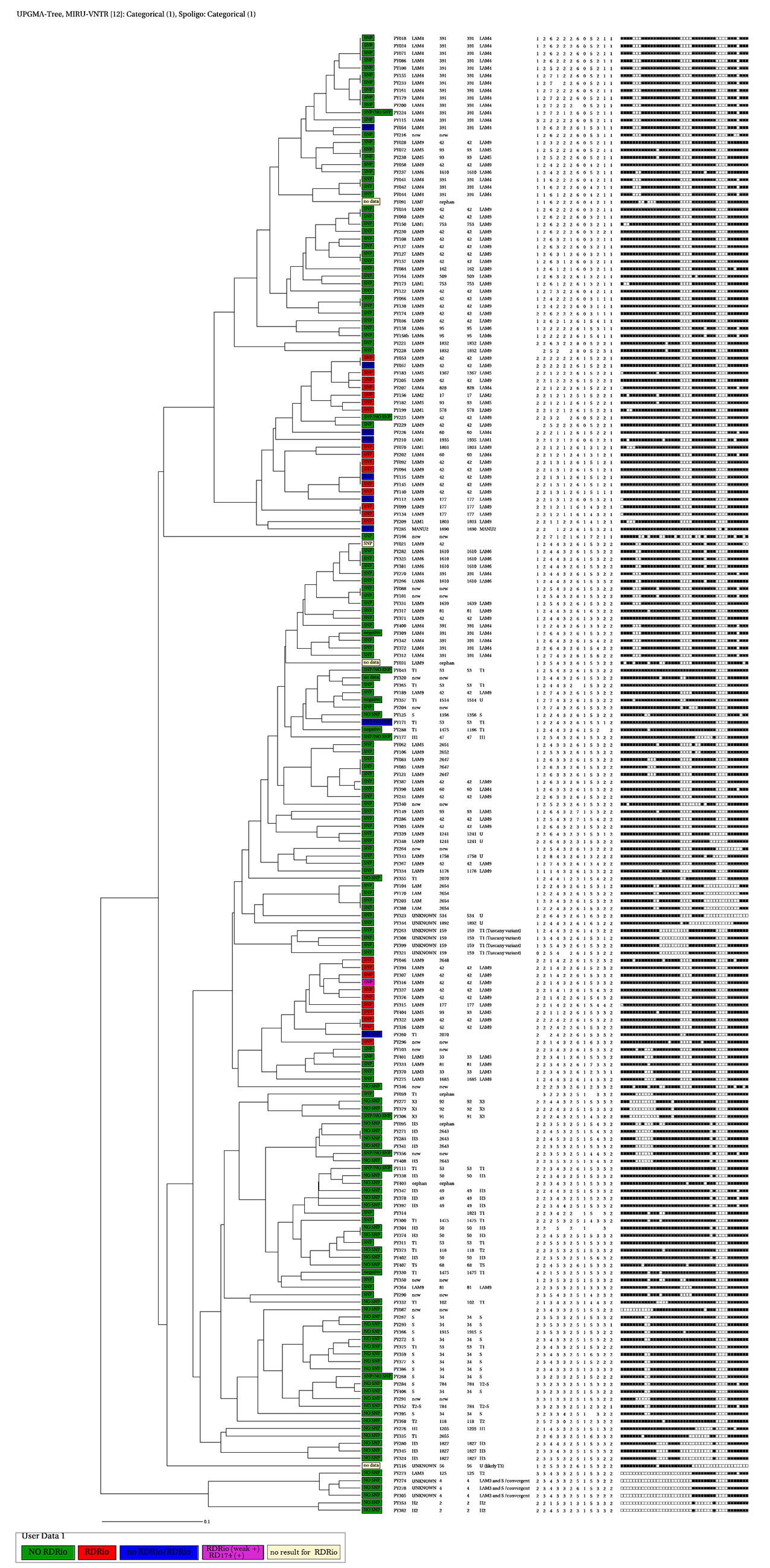

Supplement: Supplementary file 5 — Figure S5. Computer-generated UPGMA dendrogram based on combined 12 loci MIRU-spoligotyping analysis of selected strains from Paraguay, From left to right i) Boxes colors: in red RDRio strains, in green no-RDRio strains, in blue mixed RDRio/no-RDRio, PY316 colored in purple indicating a seemingly weak positive result for RDRio deletion and positive for RD174 deletion. Boxes identification: SNP or NO SNP refers to Ag85C SNP positive or negative strains respectively, ii) strain identification, iii) SITVITWEB classification iv) SIT form SITVITWEB, v) SIT from SpolDB4, vi) SpolDB4 classification, vii) 12 loci MIRU profile (MIRU02, MIRU04, MIRU40, MIRU10, MIRU16, MIRU20, MIRU23, MIRU24, MIRU26, MIRU27, MIRU31, MIRU39, MIRU40), viii) spoligotyping profile. (TIF 1164 kb) [file 12866_2019_1479_MOESM5_ESM.tif]

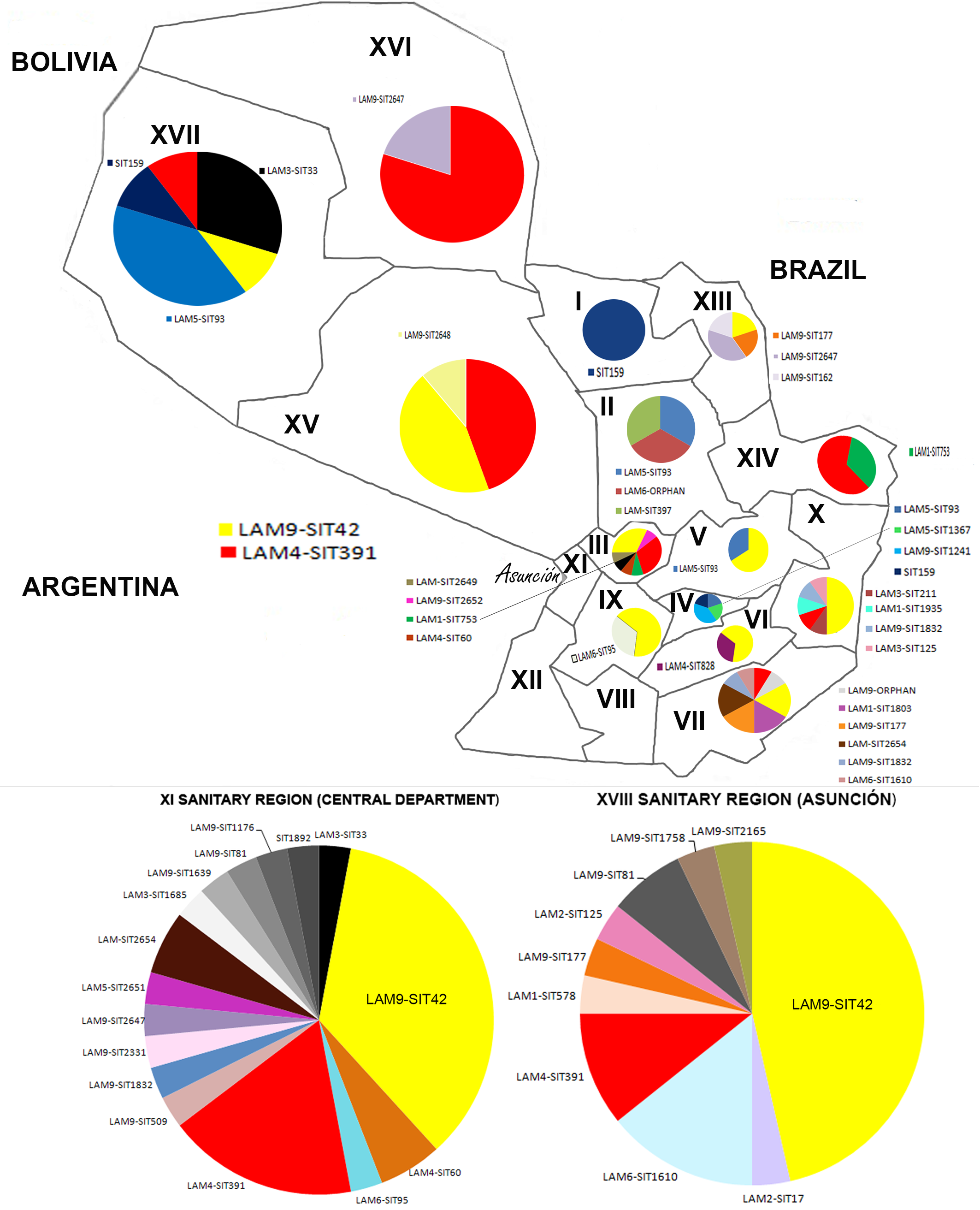

Supplement: Supplementary file 6 — Figure S6. Distribution of the LAM sub-families in each sanitary region according to patient’s residence as notified to the National TB Control Program. (TIF 1222 kb) [file 12866_2019_1479_MOESM6_ESM.tif]

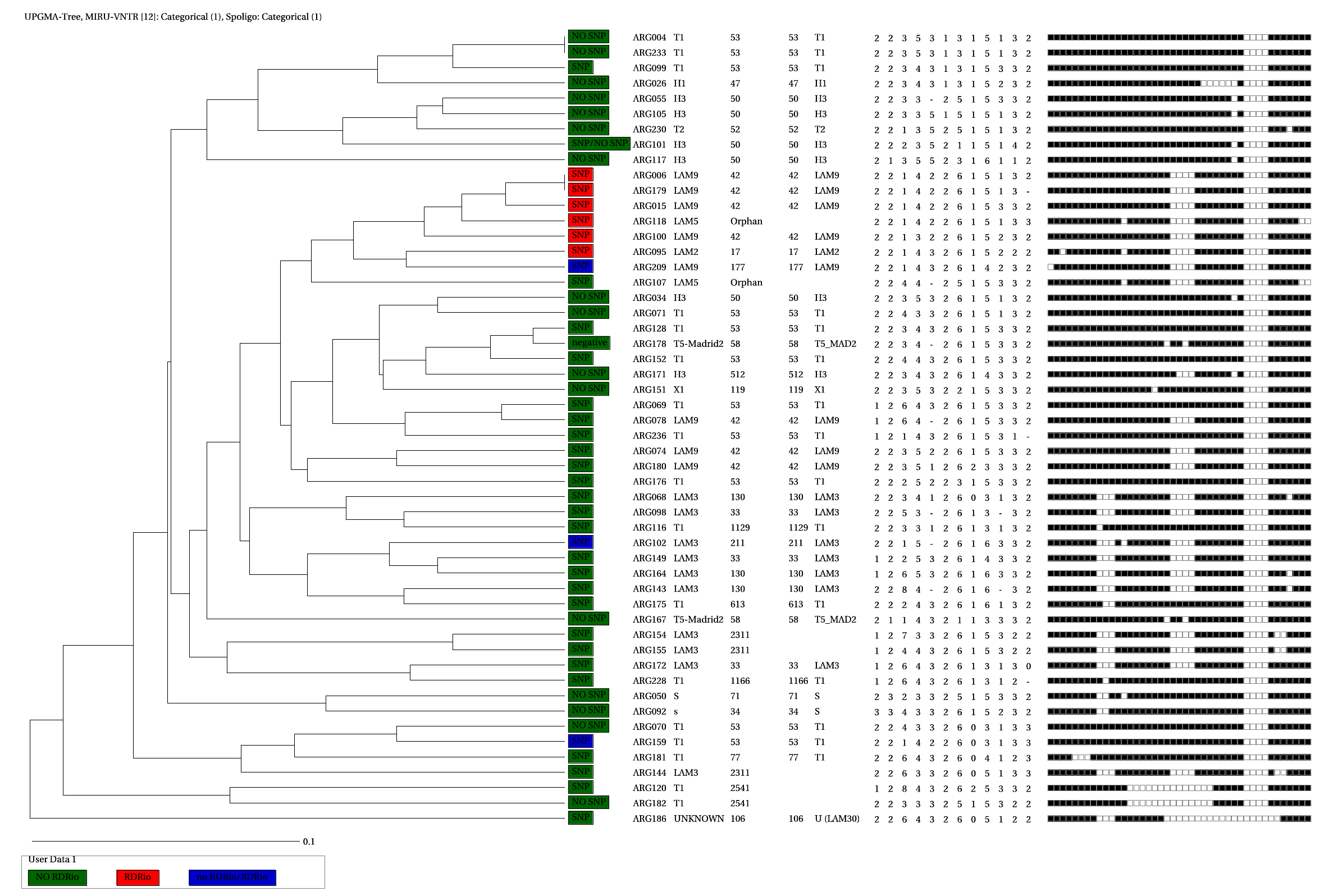

Supplement: Supplementary file 7 — Figure S7. Computer-generated UPGMA dendrogram based on combined 12 loci MIRU-spoligotyping analysis of selected strains from Buenos Aires-Argentina, From left to right i) Boxes colors: in red RDRio strains, in green no-RDRio strains, in blue mixed RDRio/no-RDRio, Boxes identification: SNP or NO SNP refers to Ag85C SNP positive or negative strains respectively, ii) strain identification, iii) SITVITWEB classification iv) SIT form SITVITWEB, v) SIT from SpolDB4, vi) SpolDB4 classification, vii) 12 loci MIRU profile (MIRU02, MIRU04, MIRU40, MIRU10, MIRU16, MIRU20, MIRU23, MIRU24, MIRU26, MIRU27, MIRU31, MIRU39, MIRU40), viii) spoligotyping profile. (TIF 422 kb) [file 12866_2019_1479_MOESM7_ESM.tif]

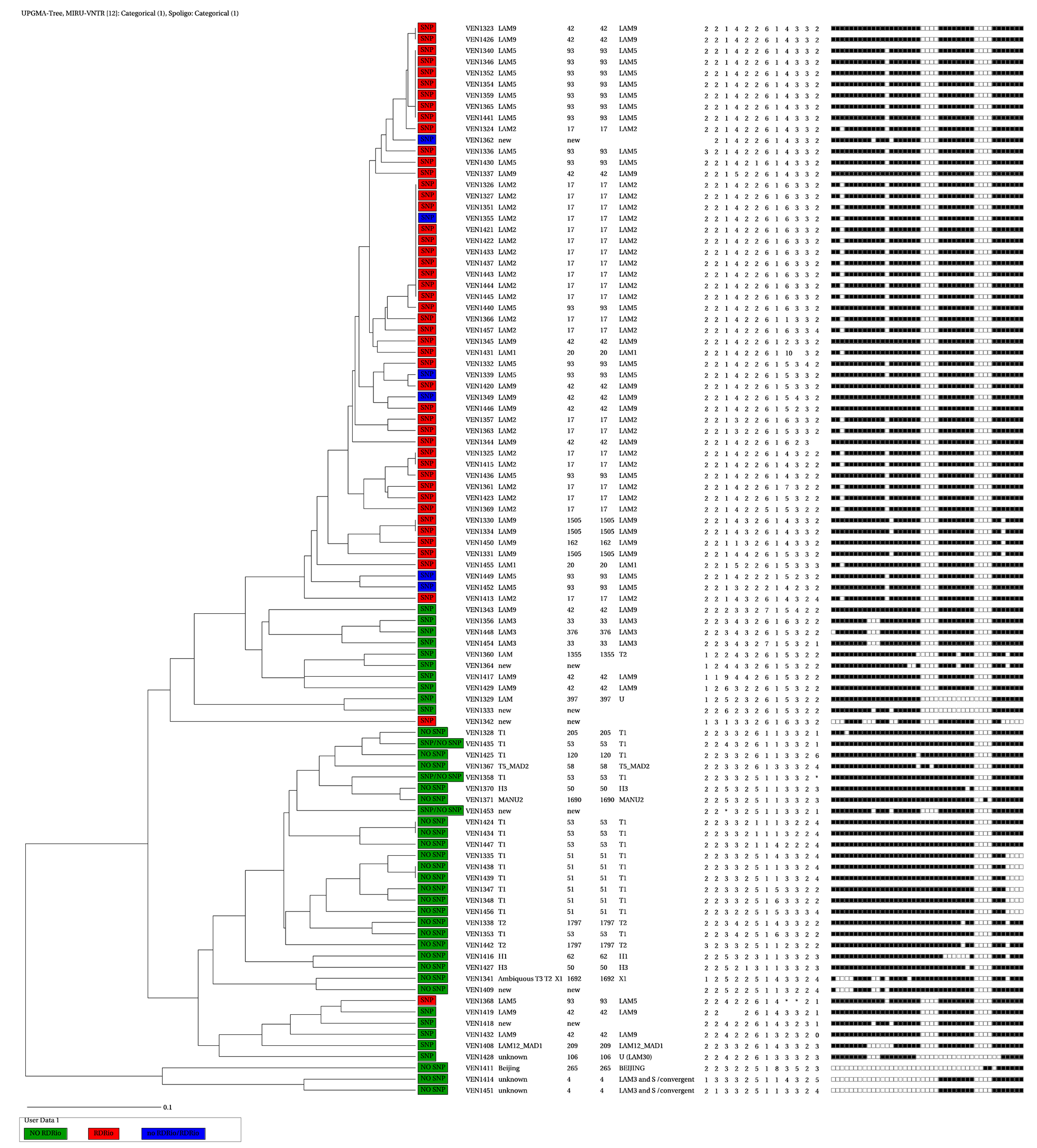

Supplement: Supplementary file 8 — Figure S8. Computer-generated UPGMA dendrogram based on combined 12 loci MIRU-spoligotyping analysis of selected strains from Caracas-Venezuela, From left to right i) Boxes colors: in red RDRio strains, in green no-RDRio strains, in blue mixed RDRio/no-RDRio, Boxes identification: SNP or NO SNP refers to Ag85C SNP positive or negative strains respectively, ii) strain identification, iii) SITVITWEB classification iv) SIT form SITVITWEB, v) SIT from SpolDB4, vi) SpolDB4 classification, vii) 12 loci MIRU profile (MIRU02, MIRU04, MIRU40, MIRU10, MIRU16, MIRU20, MIRU23, MIRU24, MIRU26, MIRU27, MIRU31, MIRU39, MIRU40), viii) spoligotyping profile. (TIF 1017 kb) [file 12866_2019_1479_MOESM8_ESM.tif]
